# Complete Genome Sequences of Bacteriophages Kaya, Guyu, Kopi, and TehO, Which Target Clinical Strains of Pseudomonas aeruginosa

**DOI:** 10.1128/MRA.01043-21

**Published:** 2021-12-02

**Authors:** Belinda Loh, Xiaoqing Wang, Xiaoting Hua, Junhan Luo, Tanye Wen, Liwei Zhang, Long Ma, Prasanth Manohar, Ramesh Nachimuthu, Ian Grainge, Yunsong Yu, Sebastian Leptihn

**Affiliations:** a Department of Biological Sciences, Xi’an Jiaotong-Liverpool University, Suzhou, PR China; b Zhejiang University-University of Edinburgh (ZJU-UoE) Institute, Zhejiang University, International Campus, Haining, Zhejiang, PR China; c Department of Infectious Diseases, Sir Run Run Shaw Hospital, Zhejiang University School of Medicine, Hangzhou, Zhejiang, PR China; d Antibiotic Resistance and Phage Therapy Laboratory, School of Biosciences and Technology, Vellore Institute of Technology (VIT), Vellore, Tamil Nadu, India; e School of Environmental and Life Sciences, The University of Newcastle, Callaghan, New South Wales, Australia; f College of Medicine & Veterinary Medicine, University of Edinburgh Medical School, Edinburgh, United Kingdom; DOE Joint Genome Institute

## Abstract

Pseudomonas aeruginosa is a major public health concern, as drug-resistant strains increase mortality in hospital-acquired infections. We report the isolation and complete genome sequences of four lytic bacteriophages that target clinical multidrug-resistant P. aeruginosa strains.

## ANNOUNCEMENT

Pseudomonas aeruginosa is an important nosocomial opportunistic pathogen that is able to live in a wide range of environments (1). The motile rod-shaped bacterium can cause lethal infections, such as sepsis in immunocompromised hosts and hospitalized patients (e.g., burn wounds), and infects a wide range of organs, including the lungs, urinary tract, and kidneys. Some strains of P. aeruginosa exhibit extensive drug resistance to available antibiotics, and the species has hence been listed as a priority 1 pathogen by the WHO (2, 3). Therefore, novel antibiotics or clinical therapeutic options are needed. One strategy is the use of therapeutic bacteriophages (4, 5). Here, we report the complete genome sequences of four lytic bacteriophages (Kaya, Guyu, Kopi, and TehO) that have been isolated using clinical multidrug-resistant strains of P. aeruginosa.

Water was collected in January 2020 from a river in Haining, China (120.605111°E, 30.481146°N). The water was filtered (pore size, 0.45 μm) before phage enrichment using cultures of P. aeruginosa. P. aeruginosa host strains were grown in lysogeny broth (LB) at 37°C overnight with agitation; the strains used to isolate each phage are provided in Table 1. Phages were obtained from clear single plaques and grown in the presence of the bacterial host in LB overnight. Bacterial cells were removed by centrifugation, and the supernatant was filtered through a 0.22-μm membrane (6). Nucleic acids were extracted using the Biomed virus rapid DNA/RNA kit (Beijing, China) according to the manufacturer’s instructions. Sequencing libraries were prepared using the NEBNext Ultra II DNA library prep kit for Illumina, and the genomes were sequenced using the Illumina HiSeq platform. The average read length obtained was 150 bp. The assembly pipeline Unicycler v0.4.8 (7) was used to conduct quality control of raw reads, assemble the genomes, and determine the completion of the assembled genomes. Genome annotation was completed using the CPT Galaxy and Web Apollo interfaces (8). tRNAs were predicted using ARAGORN v2.36 (9) and tRNA-scan-SE v2.0 (10). Open reading frames (ORFs) were predicted using GeneMarkS v4.28 (11), Glimmer v3.0 (12), and MetaGeneAnnotator v1.0 (13) and were then manually validated using BLAST v2.9.0 searches (14) against the NCBI nonredundant and Swiss-Prot databases (15). Pairwise nucleotide alignments between the phages were evaluated using NCBI blastn. Default parameters were used unless stated otherwise.

**TABLE 1:** Characteristics of Pseudomonas phage genomes

Isolate	Pseudomonas host strain	Total no. of reads (forward/reverse)	Genome coverage (×)	Genome length (bp)	GC content (%)	No. of ORFs	Accession no.	
							GenBank	SRA
Kaya	2081	11,290,334	2.56	43,067	54	60	MZ927745.1	SRR16248205
Guyu	2072	13,776,770	89.19	43,141	55	56	MZ927746	SRR16248204
Kopi	2072	7,620,976	142.96	42,820	53	55	OK330455.1	SRR16248203
TehO	2081	8,705,734	86.39	43,015	54	56	OK330456.1	SRR16248202

**FIG 1:**
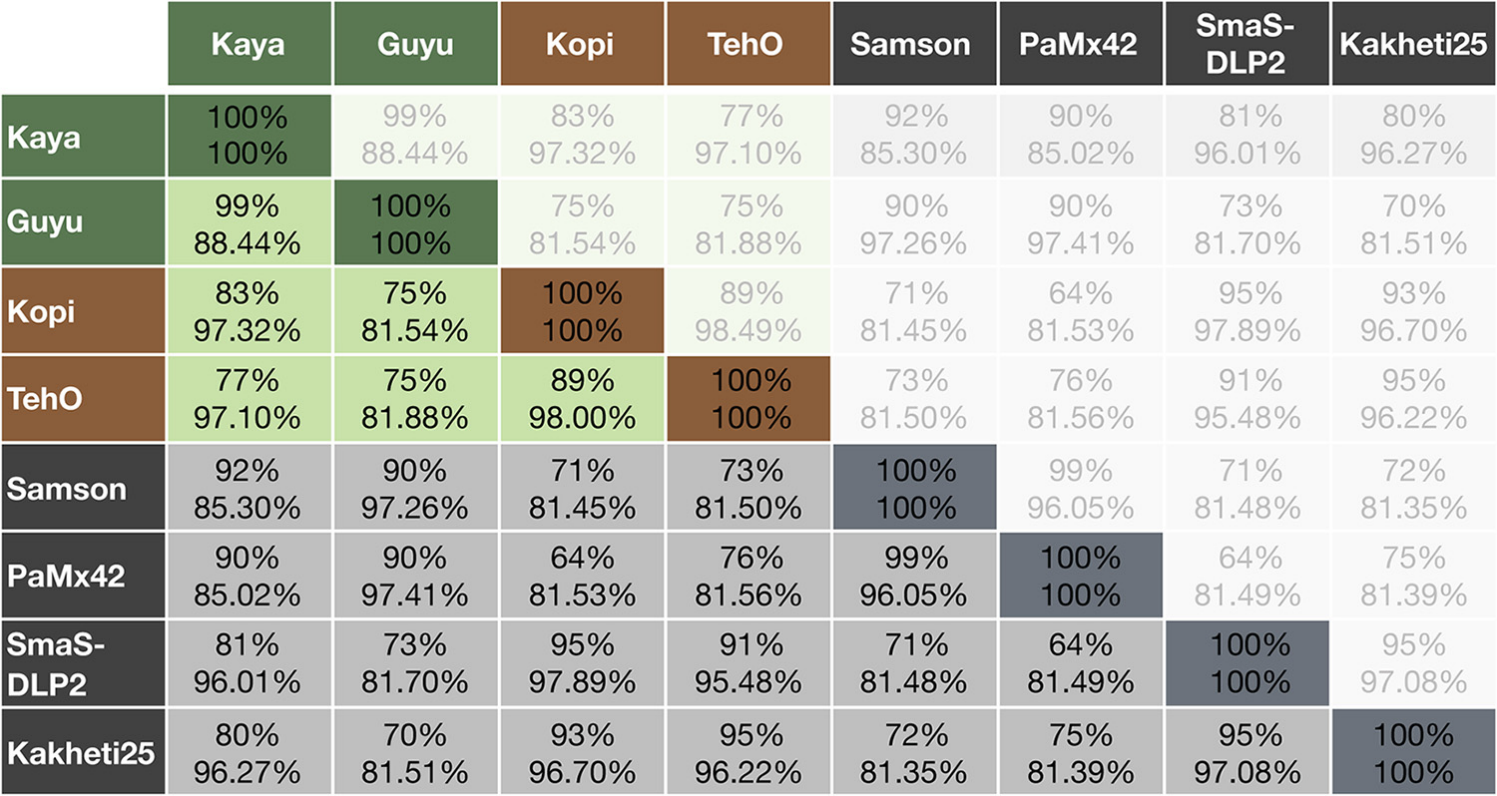
Genome sequence coverage (top number in each cell) and nucleotide identity (bottom number) of Pseudomonas phages with their closest relatives. The green and brown boxes indicate phages from this study. The gray boxes indicate phages from other studies: Samson (*Xanthomonas* phage; GenBank accession number MN062187), PaMx42 (Pseudomonas phage; JQ067092), SmaS-DLP2 (*Stenotrophomonas* phage; KR537871), and Kakheti25 (vB_Pae-Kakheti25) (Pseudomonas phage; JQ307387).

The characteristics of all four phage genomes are listed in Table 1. The phages are novel but are close relatives of each other, with their genes showing the mosaicism typical of bacterial viruses (Fig. 1). No genes were found to encode toxins or antibiotic resistance factors according to blastn searches against the Bacterial Virulence Factor Database (VFDB) (16). The phages were categorized as lytic using PhageAI (17). The most closely related phages of Kaya and Guyu are *Xanthomonas* phage Samson (GenBank accession number MN062187) and Pseudomonas phage PaMx42 (JQ067092), with genome coverage between 90% and 92% at sequence identities between 85% and 97%. Kopi and TehO are most closely related to *Stenotrophomonas* phage vB_SmaS-DLP2 (KR537871) and Pseudomonas phage vB_Pae-Kakheti25 (JQ307387), with sequence coverage between 91% and 95% at 95.48% to 97.89% sequence identity. With these sequence similarities, Kaya, Guyu, Kopi, and TehO are predicted to be *Siphoviridae* of the order *Caudovirales*.

### Data availability.

The sequencing data for bacteriophages Kaya, Guyu, Kopi, and TehO are available in GenBank under BioProject accession number PRJNA751744. The accession numbers for the genomes and sequencing reads are listed in Table 1.
